# A multi-resistance wide-range calibration sample for conductive probe atomic force microscopy measurements

**DOI:** 10.3762/bjnano.14.94

**Published:** 2023-11-22

**Authors:** François Piquemal, Khaled Kaja, Pascal Chrétien, José Morán-Meza, Frédéric Houzé, Christian Ulysse, Abdelmounaim Harouri

**Affiliations:** 1 Laboratoire national de métrologie et d’essais - LNE, Trappes, 78197 Cedex, France; 2 Université Paris-Saclay, CentraleSupélec, CNRS, Laboratoire de Génie Électrique et Électronique de Paris, 91192, Gif-sur-Yvette, France; 3 Sorbonne Université, CNRS, Laboratoire de Génie Électrique et Électronique de Paris, 75250, Paris, France; 4 Centre de Nanosciences et de Nanotechnologies - C2N, Université Paris-Saclay, CNRS, UMR 9001, Palaiseau, 91120, France

**Keywords:** calibration, conductive probe atomic force microscopy, measurement protocol, nanoscale, resistance reference

## Abstract

Measuring resistances at the nanoscale has attracted recent attention for developing microelectronic components, memory devices, molecular electronics, and two-dimensional materials. Despite the decisive contribution of scanning probe microscopy in imaging resistance and current variations, measurements have remained restricted to qualitative comparisons. Reference resistance calibration samples are key to advancing the research-to-manufacturing process of nanoscale devices and materials through calibrated, reliable, and comparable measurements. No such calibration reference samples have been proposed so far. In this work, we demonstrate the development of a multi-resistance reference sample for calibrating resistance measurements in conductive probe atomic force microscopy (C-AFM) covering the range from 100 Ω to 100 GΩ. We present a comprehensive protocol for in situ calibration of the whole measurement circuit encompassing the tip, the current sensing device, and the system controller. Furthermore, we show that our developed resistance reference enables the calibration of C-AFM with a combined relative uncertainty (given at one standard deviation) lower than 2.5% over an extended range from 10 kΩ to 100 GΩ and lower than 1% for a reduced range from 1 MΩ to 50 GΩ. Our findings break through the long-standing bottleneck in C-AFM measurements, providing a universal means for adopting calibrated resistance measurements at the nanoscale in the industrial and academic research and development sectors.

## Introduction

Since its introduction thirty years ago by Murrell et al. [1], conductive probe atomic force microscopy (C-AFM) has evolved into a unique and powerful technique for measuring local electrical quantities (i.e., current and resistance) at the nanoscale. In C-AFM, a micro-machined conductive probe with a sharp nanometer-sized tip acts as a top electrode brought into contact with the surface of a sample while applying a potential difference relative to a back electrode. The small currents flowing through the system are measured using a current amplifier, typically ranging from 100 fA to 10 µA for most commercially available microscopes [2–3]. By sweeping the potential difference while the tip is fixed in contact with the sample, current versus voltage (*I*–*V*) curves are acquired. *I*–*V* curves are essentially used to extract resistance values or to characterize the electric behavior of components and devices [4]. Alternatively, current variation maps are acquired at a given applied voltage by scanning the AFM tip in contact mode across a defined sample surface area [5]. Owing to its versatility and high resolution in probing the local conductivity of materials, C-AFM has been extensively used in studying semiconductors [6–7], two-dimensional materials [8–10], memristive devices [11–15], photoelectric systems [16–18], dielectric films [19–23], molecular electronics [24–29], organic and biological systems [30–34], and quantum devices [35–37]. Various technical methods have been developed in C-AFM to cope with the diversity of its applications, including advanced sensors and low-noise preamplifiers [2,38–40]. Nevertheless, quantifying the measured currents and resistances remains a bottleneck issue in C-AFM, inhibiting an effective comparison of results to comprehend experimental processes.

C-AFM measurements are prone to environmental and experimental factors that heavily affect their stability, reproducibility, repeatability, and exactness [41–42]. The formation of a humidity-induced water meniscus at the tip–sample interface, the presence of surface contamination, and thermal drifts induce significant instabilities in C-AFM measurements [42–43]. Moreover, local overheating and anodic oxidation phenomena are commonly observed in C-AFM because of highly localized electric fields at the tip apex leading to structural damage considerably affecting the measurement reliability. These effects are further amplified during scanning in contact mode due to shear forces and strong mechanical stress imposed on the tip apex [44]. Therefore, it is common to measure sudden alterations in local currents and resistances in C-AFM unrelated to the sample’s physical properties [43]. The combination of the effects above makes it difficult to quantify and reproduce the measured values in C-AFM experiments, which degrades the method’s efficiency in advancing the understanding of many processes in materials sciences and industrial developments. Despite the widely experienced difficulties, no universal solution to ensure the calibration and traceability of C-AFM measurements has been proposed in the literature. So far, only personalized custom approaches have been adopted that are restricted to specific setups or experiments [20,45].

In this paper, we propose a multi-resistance reference sample covering a wide range of values from 100 Ω to 100 GΩ, enabling a universal calibration approach to quantitative measurements in C-AFM applicable to all systems and setups.

## Results and Discussion

Our approach consists of three main steps performed in a one-month timeframe. First, we calibrate the resistors employed in the fabrication of the reference sample using probe station measurements. Second, we use C-AFM imaging to obtain resistance maps and identify the error sources associated with the imaging-mode measurements. Finally, we apply spectroscopic C-AFM measurements to extract current versus voltage (*I*–*V*) curves for each resistance value on the sample. We undertake a comprehensive analysis to compare resistance values obtained by C-AFM imaging and *I*–*V* curves measurement to define the conditions for calibrated measurements.

### Calibration sample design and fabrication

**Figure 1:**
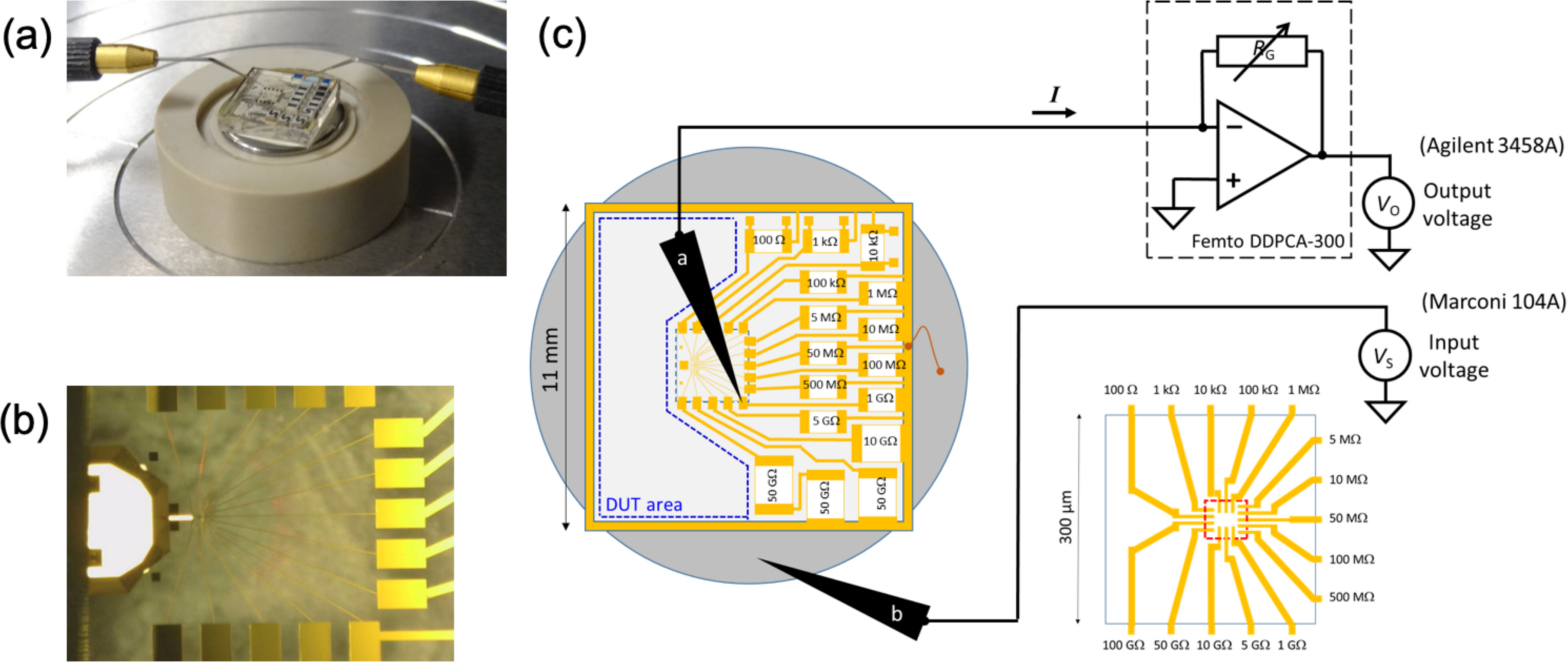
(a) Picture of the resistance reference sample connected to the probe station. (b) Top view of the sample underneath the AFM tip. (c) Schematics of the resistance measurement circuit involving two microprobes, an ultra-low current amplifier, and a digital nanovoltmeter. The first drawing shows the sample with the 15 resistive arms and a free area of around 35 mm^2^ (delimited by blue dotted lines) designed to place a device under test (DUT) with a possible connection to the peripheral gold line. The second drawing shows the central zone (delimited by a red dotted line) of the sample designed for local imaging and spectroscopic measurements in C-AFM.

The sample developed in this work consisted of a square fused silica substrate (11 mm wide, 2 mm thick), on which gold connection lines and pads were fabricated by standard photolithography, using a mask aligner (MA6, Karl Suss, Germany), and conventional deposition techniques. Following a resist (about 1 µm thick) development process, a 2 inch diameter wafer was placed in a vacuum chamber for electron beam deposition of a 200 nm thick titanium/gold layer. Subsequently, a lift-off process in acetone was employed to reveal the gold pattern. Finally, square samples were cut to match the dimensions of the measurement setup. We hand-soldered thick-film surface-mounted-device (SMD) resistors onto the connection gold pads on the surface using small soldering paste droplets (F42240, lead-free solder paste – class 5, CIF, France). The fused silica substrate was placed on a heating plate set to 270 °C, which required around 3 min to reach the melting temperature of the solder droplets (217 °C), as observed under an optical microscope. Upon cooling, 16 SMD resistors were fixed on the sample surface, creating a set of 15 resistance values, as shown in Figure 1a. The substrate was fixed onto a circular metallic plate (15 mm diameter), which acts as a back electrode connected to all resistances using a peripheral gold line and dashes of silver paste deposited on the sample edges. Each resistance was connected to an intermediate gold pad (300 µm × 470 µm) designed for microcontacting using a probe station setup, as shown in Figure 1a,b. Furthermore, the contacts were extended to the central area (60 µm × 60 µm) of the sample, forming a set of 15 small (i.e., 5 µm wide) electrode arms designed for local C-AFM imaging and spectroscopic measurements. The gold lines’ dimensions were characterized for calculating their intrinsic resistances using the gold resistivity value.

### Calibration of SMD resistors and gold lines

Before conducting C-AFM measurements, the resistance values of the SMD resistors and the gold connection lines should be determined using calibrated equipment. To this end, the intermediate gold pads were used as terminals to calibrate the corresponding resistance values relative to the back electrode. We used a probe station (Cascade Microtech MPS150) coupled to a programmable voltage source (Marconi 104A) and a high-precision ammeter to measure the resistance values of the SMD devices in an electromagnetically shielded environment under stabilized air temperature (22.9 ± 0.1) °C and relative humidity (40.7 ± 0.3)%. Two different calibrated ammeters were used depending on the range of the expected resistance values. As shown in Figure 1, a digital voltage multimeter (DVM) (Keysight 3458A) was used for the resistance range between 100 Ω and 1 GΩ, while a very low noise (fA/Hz^1/2^) current amplifier (Femto DDPCA-300) was associated with the same DVM for the upper resistance range between 1 GΩ and 100 GΩ. The DVM and the current amplifier were calibrated at the French National Metrology Institute (LNE) following the highest standards in metrology (see Supporting Information File 1, section S1).

Table 1 compares the nominal resistance values with those measured for each resistor, *R*_i,meas_, at the rectangular pads using probe station measurements with the combined uncertainties. All uncertainties in the paper are given at one standard deviation corresponding to a 68% confidence level in the case of a normal distribution [46]. All measured values were in excellent agreement with the nominal ones within the tolerance limit indicated by the manufacturer, except for the first three pads. Owing to their low values, these three resistances (*R*_1,meas_, *R*_2,meas_, and *R*_3,meas_) were corrected by accounting for the resistances of the connection line segments, *R_i_*_,seg_, in the central zone of the sample, and the resistance of the wiring, *R*_wire_, between the two probes and the DVM. Considering the dimensions of the line segments and the measured resistivity of the deposited gold lines (ρ = (31.4 ± 0.4) × 10^−9^ Ω·m), we calculated three correction resistances *R*_1,seg_ = 21.2 Ω, *R*_2,seg_ = 20.1 Ω, and *R*_3,seg_ = 22.4 Ω for the first three pads, respectively. The measured value of the supplementary resistance due to the wiring (including the resistance of the two probes and the cable resistances) was determined at *R*_wire_ = 1.8 Ω (see Supporting Information File 1, section S2).

**Table 1:** Nominal (*R_i_*_,nom_) and measured (*R_i_*_,meas_) values for the 15 pads and combined uncertainties *u_i_* in relative values (%). The uncertainties are given at one standard deviation. The tolerance on the values of the mounted resistors and the measurement date are given.

*i* (pad index)	*R_i_*_,nom_ (Ω) (resistor)	Tolerance (%)	*R*_i,meas_ (Ω) (pad)	*u*_i_ (%)

1	1 × 10^2^	0.5	1.672 × 10^2^	0.03
2	1 × 10^3^	1	1.068 × 10^3^	0.03
3	1 × 10^4^	0.05	1.007 × 10^4^	0.03
4	1 × 10^5^	0.1	1.000 × 10^5^	0.03
5	1 × 10^6^	1	1.000 × 10^6^	0.03
6	5 × 10^6^	1	5.011 × 10^6^	0.03
7	1 × 10^7^	1	0.998 × 10^7^	0.03
8	5 × 10^7^	1	4.975 × 10^7^	0.03
9	1 × 10^8^	1	0.998 × 10^8^	0.03
10	5 × 10^8^	5	5.043 × 10^8^	0.06
11	1 × 10^9^	10	1.000 × 10^9^	0.09
12	5 × 10^9^	10	4.610 × 10^9^	0.13
13	1 × 10^10^	30	0.972 × 10^10^	0.13
14	5 × 10^10^	30	3.611 × 10^10^	0.17
15	1 × 10^11^	30	0.784 × 10^11^	0.17

The combined uncertainty values in Table 1 were calculated using the root-sum-square method (RSS) from uncertainties related to the sample, the environmental conditions, the measurement circuit, and the measurement repeatability. The uncertainties were estimated using the reference evaluation methods [46]. The major uncertainty components originated from the sample temperature and voltage effects, ranging from 1.1 parts in 10^3^ to 1 part in 10^4^ with decreasing resistance values. The other main uncertainties did not exceed 4 parts in 10^4^, which were related to the calibrations of the measurement instruments (particularly the current amplifier gain), the leakage resistances, and the measurement noise (see Supporting Information File 1, sections S3 and S4).

### Resistance values in C-AFM imaging mode

Following the calibration of the SMD resistors, C-AFM imaging measurements were conducted by scanning the central zone of the sample. Experiments were performed using a Multimode 8 AFM system with a Nanoscope V controller (Bruker, USA) operated in contact mode with CDT-FMR diamond-coated probes (Nanosensors, USA). Resistance maps (512 × 512 pixels) were recorded using a recently developed custom-built external wide-range current measuring device (WCMD), connected to the AFM system operating under ambient environmental conditions (no shielding and no air conditioning system). The WCMD device consists of a current amplifier with an automatic gain regulation. It allows for, under usual AFM scanning conditions, current and resistance mapping as well as *I*–*V* spectroscopy over a wide range of current measurement (from 100 µA to less than 100 fA) (see Supporting Information File 1, section S1). Previous experiments have shown diamond-coated tips to be most suitable for imaging gold surfaces in ambient air. A DC bias voltage of 1 V was applied to the sample, while the scanning speed was set to 12 µm·s^−1^ and the scan orientation was parallel to the cantilever’s central axis.

**Figure 2:**
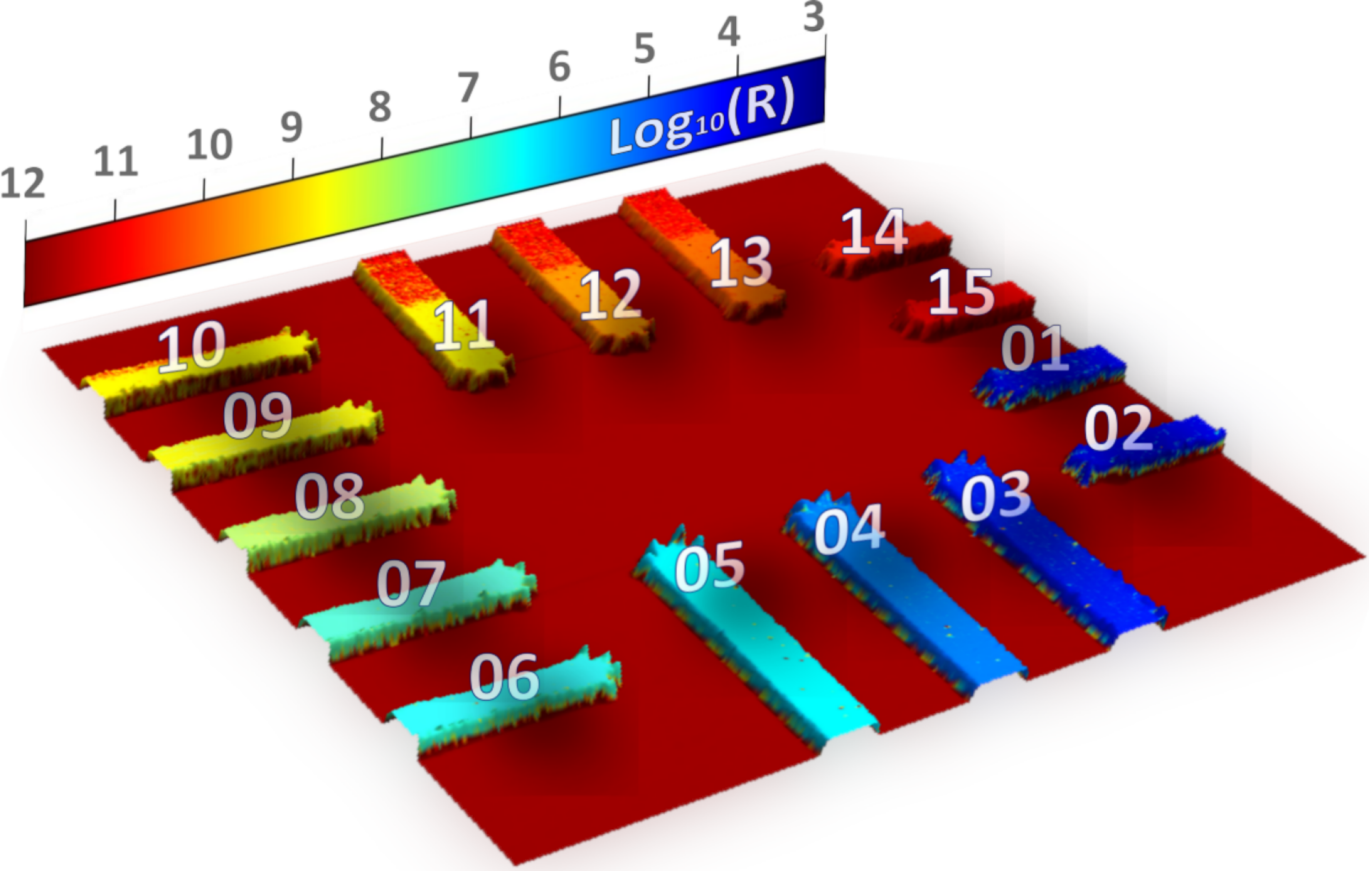
Resistance map of the sample’s central zone (60 µm × 60 µm) imaged by C-AFM. Numbers refer to the index *i* of the resistance arms. The color rendering refers to the measured resistance values given in decimal logarithm scale.

The resistance map in Figure 2 was acquired over the central zone of the sample, showing 15 electrode arms corresponding to the end of the gold connection lines linked to the intermediate gold pads previously measured in Table 1. The imaging result shows a distinguishable resistance contrast for the values expected between 10 kΩ and 100 GΩ, which validates the applicability of the developed sample for the calibration of C-AFM measurements in scanning mode. To extract quantitative values comparable to those listed in Table 1, the surface of each electrode was individually imaged at different locations using the same operating parameters, that is, scan speed, scan orientation, applied force, and bias voltage. A histogram was extracted for each resistance map, and the data were fitted to Gaussian distributions. The results showed that the mean value of measured resistances deviates significantly from the expected value in Table 1 by more than 100% for the first three electrode arms *i* = 1 to *i* = 3 (i.e., 100 Ω, 1 kΩ, and 10 kΩ). In this case, the significant deviation was attributed to the high resistance of the AFM tip (ca. 10 kΩ, nominal value from the manufacturer), which prevents a correct measurement of small resistance values. For the remaining electrode arms *i* = 4 to *i* = 15 (i.e., 100 kΩ to 100 GΩ), the measured values from the resistance maps deviated by 20% to 28% compared to those determined in Table 1. This error was partly related to an erroneous reading from the AFM controller unit, which systematically added an offset to the measured values, as identified by injecting external test DC voltage signals to the controller. Thus, further measurements were conducted by shortcutting the AFM controller and recording resistance values measured directly by the WCMD device. Nonetheless, a remaining deviation of the resistance values obtained in C-AFM imaging mode relative to the values in Table 1 was still observed of the order of 8%.

### Resistance values from C-AFM *I*–*V* curves

To comprehend the origin of this remaining error, we proceeded into removing any possible contamination of the tip apex by repeatedly scanning over a fixed line (typically a few tens of nanometers) on the sample surface (i.e., by disabling the slow-scan axis). The effective contamination removal was associated with a stable measurement of a minimal resistance value. Then, we positioned the tip at a fixed location in contact with the electrode’s surface with an applied force of 900 nN to extract *I*–*V* curves by sweeping the applied voltage between −1 V and +1 V. This approach mitigates the difficulties related to surface contamination on the gold electrodes during scanning. Resistance values for each electrode arm were determined from the slopes of the *I*–*V* straights using a regression model. For each value, the coefficient of determination (*R*^2^) was equal to 1 (see Supporting Information File 1, section S5 and Figure S1). The results were globally found within a 2.5% deviation relative to the resistance values in Table 1.

**Figure 3:**
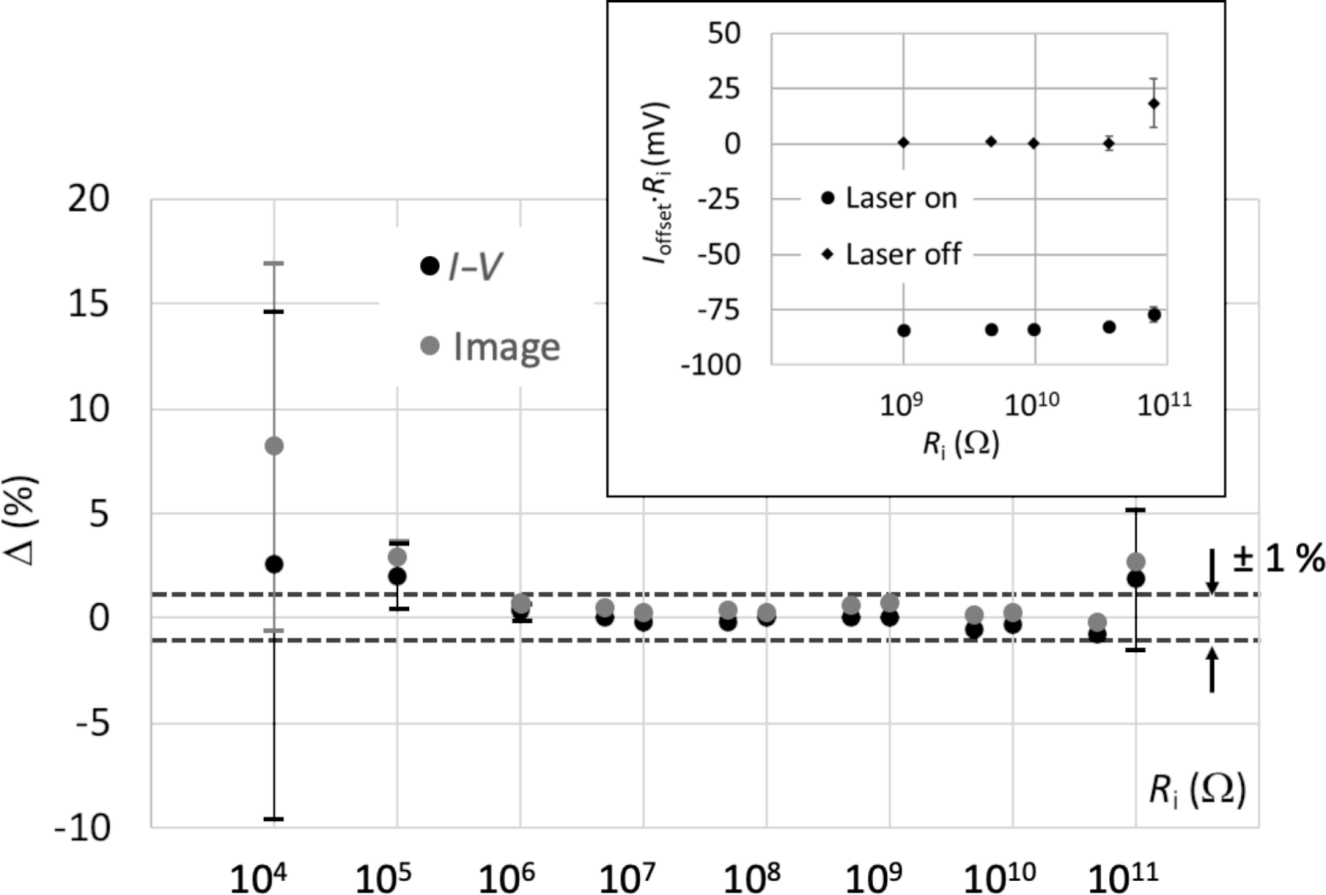
Relative deviations Δ = (*R_i_*_,AFM_ – *R_i_*_,cal_)/*R_i_*_,cal_ in percent from imaging (full grey circles) and from *I*–*V* curves (full black circles). Values for 10 kΩ, 100 kΩ, and 1 MΩ were corrected to take into account the measured tip resistance (6591 Ω). The error bars denote the uncertainties calculated from the RSS method from the total uncertainties due to the resistance reference (reported in Table 1) and the measurement repeatability. In the insert, the products *I*_offset_·*R_i_* are given in millivolts, where *I*_offset_ denotes the current offset observed at zero bias voltage on the *I*–*V* curves, when the AFM laser is turned on (full circles) and off (full diamonds), for the highest resistances. The error bar (only visible for 10^11^ Ω) refers to the standard deviation of data (repeatability).

In comparison, an excellent agreement (within 1%) was obtained for the specific range of 1 MΩ and 50 GΩ, as shown in Figure 3. The resistance values for the electrode arms *i* = 3, 4, and 5 (i.e., 10 kΩ, 100 kΩ, and 1 MΩ, respectively) were corrected by accounting for the tip resistance, which was measured on a copper film at *R*_tip_ = 6591 Ω with a relative uncertainty of 1% (conservative value). It is worth noting that, for higher resistance values up to 100 GΩ, the correction accounting for the tip resistance value becomes largely insignificant. Despite the reduced uncertainty for the resistance values determined from the *I*–*V* curves, those obtained from the imaging results still showed a non-negligible deviation. In addition, we noticed that all *I*–*V* straights did not pass through zero, which introduced a shift in the measured currents leading to an increase in the resistance values by a constant amount of (+8 ± 1)%, which agrees very well with the deviations observed from the image values (taken at a bias voltage of +1 V).

The origin of *I*–*V* curves not going through zero is commonly associated with photovoltaic effects, which was indeed validated by the disappearance of this observation when the laser of the AFM setup was switched off. Although, a photovoltaic effect might be intriguing in current measurements on gold pads, this observation was systematically made for the *I*–*V* curves measured on all gold electrode arms. Through further investigations, we were able to associate this observation with the use of worn AFM diamond tips, especially formed by a p-type diamond coating on a highly doped n-type Si core. Thus, the photovoltage effect observed in our paper is solely related to the tip apex and does not depend on the measured sample. We were able to confirm this aspect by running *I*–*V* curves using new probes with intact apexes, which showed no shift around zero even with the AFM laser on. This effect is currently under thorough investigations for a future publication. Accordingly, a new set of images was acquired for the electrodes *i* = 3 to *i* = 15 at two bias voltages of +1 V and −1 V, and the corresponding resistance value was determined by their mean value. For each electrode, this imaging protocol was repeated at three to five zones to enhance statistical values. The final resistance of an electrode corresponded to the average value of the three to five measurements. Figure 3 shows an excellent agreement between the resistance values obtained from C-AFM images and those from *I*–*V* curves with a maximum global deviation of 1%. However, the electrode arm 10^4^ Ω (*i* = 3) showed 5.7% deviation, which is well within the corresponding uncertainty.

Our findings show that the multi-resistance reference sample developed in this work enables a universal calibration of C-AFM measurements in both imaging and spectroscopic (i.e., *I*–*V* curves) modes with a 1% achievable relative uncertainty level in the range between 10^6^ Ω and 5 × 10^10^ Ω. The protocols adopted in this study highlight several routes for further improvements. Using platinum as metallic material instead of gold for the small electrode arms would help reduce surface contamination-related issues. Consequently, measuring the lowest resistance values would become accessible using low-resistance metallic probes (e.g., Pt-coated or full bulk Pt probes). However, using such probes will require limiting the current (typically 100 µA) to avoid excessive Joule heating within the nanocontact.

## Conclusion

We have designed a multi-resistance wide-range reference for calibrating the complete C-AFM measurement circuit over a resistance range from 100 Ω to 100 GΩ. A set of operating protocols have been demonstrated for measuring resistance in C-AFM within the range from 10 kΩ to 100 GΩ with deviations lower than 2.5% relative to values calibrated at the macroscale using probe station measurements. The design of the proposed calibration sample features access to a wide range of resistance values (nine decades) within a single AFM scan, calibration of these resistances at the macroscale using a probe station, compatibility with any commercially available AFM system, and the possibility of positioning a device under test (DUT) on the reference sample. Further efforts are underway to develop another sample version featuring easier access to C-AFM measurements of the lowest resistances (from 100 Ω to 10 kΩ) and an expanded resistance range up to 1 TΩ. The outcome of the present work is expected to promote the applicability of C-AFM for the local measurements of DC resistances and currents at the nanoscale, which constitutes an essential requirement for coping with the ever-increasing shrinkage of technological devices. It is worth noting that the authors are closely working with the International Electrotechnical Commission (IEC-TC113) for the creation of documentary standards regarding resistance measurements in C-AFM.

## Supporting Information

File 1 Additional experimental information.
