# Supplementary material for: A multi-resistance wide-range calibration sample for conductive probe atomic force microscopy measurements
Source: Beilstein J Nanotechnol. 2023 Nov 22;14:1141–8. doi: 10.3762/bjnano.14.94 (PMC10682512; doi:10.3762/bjnano.14.94)
Supplement: File 1 — Additional experimental information. [file Beilstein_J_Nanotechnol-14-1141-s001.pdf]

BEILSTEIN JOURNAL OF NANOTECHNOLOGY

Supporting Information

for

A multi-resistance wide-range calibration sample for conductive probe atomic force microscopy measurements

François Piquemal, Khaled Kaja, Pascal Chrétien, José Morán-Meza, Frédéric Houzé, Christian Ulysse and Abdelmounaim Harouri

Beilstein J. Nanotechnol. **2023,** *14,* 1141–1148. doi:10.3762/bjnano.14.94

Additional experimental information

License and Terms: This is a supporting information file under the terms of the Creative Commons Attribution License (https://creativecommons.org/licenses/by/4.0). Please note that the reuse, redistribution and reproduction in particular requires that the author(s) and source are credited and that individual graphics may be subject to special legal provisions.
The license is subject to the *Beilstein Journal of Nanotechnology* terms and conditions: (https://www.beilstein-journals.org/bjnano/terms)

S1 – Instrument Calibration

The DVM Keysight 3458A has an inherent accuracy of few ppm for DC voltage, resistance, and current measurements, which makes it an extremely valuable asset in metrology labs. Both the DVM and the Femto amplifier were calibrated at the French National Metrology Institute (LNE), following the highest standards in metrology (see for example the Key Comparison Data Base of the BIPM: https://www.bipm.org/kcdb/cmc/quick-search). The custom-built WCDM device was calibrated in-house using discreet resistances, yielding an uncertainty of 1% for resistances in the range between 100 Ω and 10^9 Ω, and 10%–20% for resistances in the range between 10^{10} Ω and 10^{12} Ω. It is worth highlighting that, to our knowledge, there are no readily existing calibrated devices for resistance measurements in C-AFM.

S2 – Resistance Correction of the Connection Lines

To determine the resistance of the connection line segments, we proceeded as follows: We performed four-wire measurements between the intermediate gold pads (300 µm × 470 µm) and the ground for the first three arms (100 Ω, 1 kΩ, and 10 kΩ). We calculated the resistivity ($\rho = (31.4 \pm 0.4)\cdot10^{-9}$ Ω·m) using the measured resistance values and dimensions (width and thickness) of the lines. Then, we measured the dimensions of the lines between the intermediate gold pads and the electrodes. Based on the calculated resistivity value and the line dimensions, we calculated their resistance values to make the appropriate corrections.

S3 – Measurement Protocol for Reference Values in Table 1

We collected 100 measurement data for each resistance value ranging from 100 Ω to 1 GΩ using the high-precision DVM. This leads to standard deviations not exceeding 4 parts in 10^5. Moreover, we measured a single *I*–*V* curve for each resistance in the range between 1 GΩ and 100 GΩ by sweeping the voltage between −1 V and +1 V with steps of 50 mV. This leads to a standard deviation on the slope of less than 8 parts in 10^5. These standard deviations reflect the measurement noise. In metrology, we consider this uncertainty as a type-A standard uncertainty (Joint Committee for Guides in Metrology - JCGM. Evaluation of Measurement Data — Guide to the Expression of Uncertainty in Measurement. JCGM 100 2008. https://www.bipm.org/en/publications/guides/gum.html).

S4 – Uncertainty Components (Sample Temperature and Voltage Effect)

The uncertainty components originating from the sample's temperature and voltage effects were estimated from the temperature coefficient and the voltage effect coefficient given by the manufacturers. In fact, voltage effects correspond to power effects, that is, self-heating of the resistors, generally correlated to the temperature coefficient of that resistor. The temperature coefficient ranges from 10^{-4}/K in relative value for 100 Ω up to $2\cdot10^{-3}$/K for 100 GΩ. The voltage coefficient ranges from $5\cdot10^{-4}$/V for 100 Ω up to $2\cdot10^{-3}$/V for 100 GΩ. A rectangular distribution was considered in both cases. The uncertainty component corresponding to the calibration of the current amplifier gain is mainly dominated by the calibration uncertainty of the resistance standards used to calibrate the gain, that is, three resistances of 1 GΩ, 10 GΩ, and 100 GΩ, with a calibration uncertainty of 2.6 parts in 10^4 (one standard deviation).

S5 – *I*–*V* Curves Analysis for Resistance Assessment

I–*V* curves were analyzed with a custom-made MATLAB-based analysis software to extract resistance measurements from the slope of *I*–*V* curves using a regression model. For each calculated mean value,

the value of R^2 (coefficient of determination) was equal to 1. Figure S1 shows the *I–V* curves for nominal resistances values of 1 MΩ, 10 MΩ, 100 MΩ, 1 GΩ, and 10 GΩ.

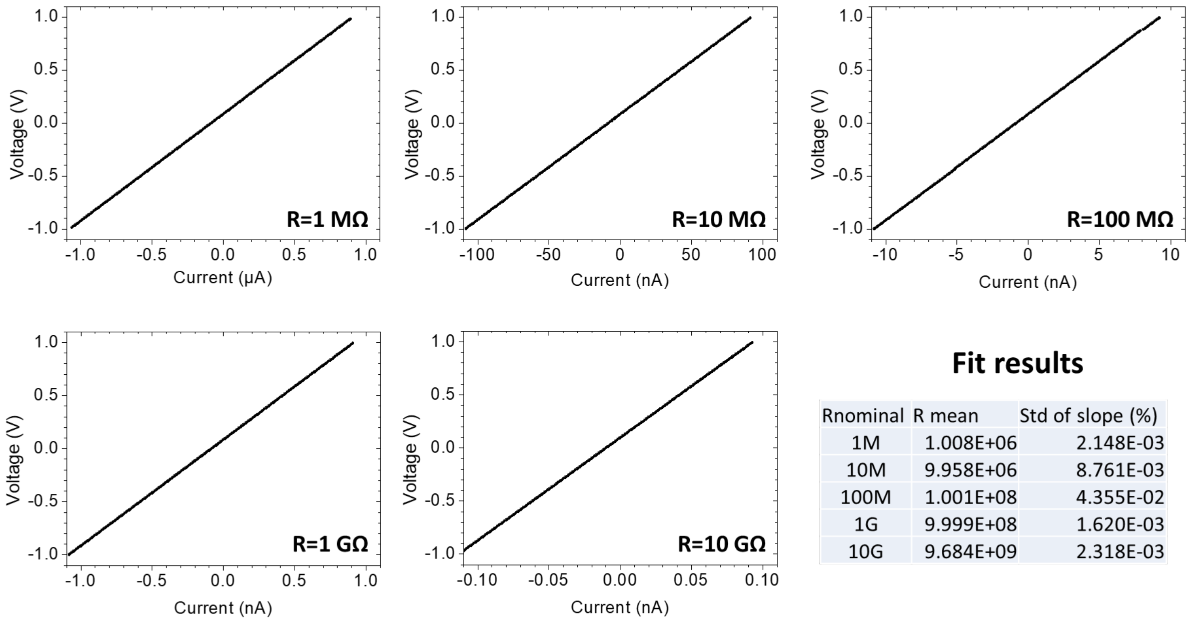

Fit results

Rnominal	R mean	Std of slope (%)
1M	1.008E+06	2.148E-03
10M	9.958E+06	8.761E-03
100M	1.001E+08	4.355E-02
1G	9.999E+08	1.620E-03
10G	9.684E+09	2.318E-03

Figure S1: Representative examples of the *I–V* curves measured on the different resistance pads. The last panel shows the fit results to determine the resistance values from the slopes of the *I–V* curves.
